# Local recurrence of gastric cancer after total gastrectomy: an unusual presentation

**DOI:** 10.1186/1471-2482-12-S1-S28

**Published:** 2012-11-15

**Authors:** Bruno Martella, Fabrizio Cardin, Renata Lorenzetti, Claudio Terranova, Bruno Amato, Carmelo Militello

**Affiliations:** 1 Department of Molecular Medicine, University of Padua, Italy; 2 Department of Surgical and Gastroenterological Sciences, University of Padua, Italy; 3 University of Naples Federico II - Department of General Surgery, Italy

## Abstract

A 71 years old Italian man had type 3 gastric cancer of the greater curvature. Total gastrectomy with splenectomy and D2 lymph node dissection were performed. After discharge chemotherapy ELF regimen was administred for 6 months. After 16 months from the operation a local recurrence was discovered by CT scan. Surgical en-bloc resection was performed removing pancreatic tail, splenic colic flexure and a portion of left diaphragm. Histological examination confirmed local recurrence of gastric adenocarcinoma infiltrating pancreas, colon and diaphragm with lymph node metastasis.

## Introduction

In Western countries gastric cancer still represents a disabling disease: unfortunately late diagnosis is common and loco-regional recurrence rate after surgery alone is high especially in patients with advanced stage disease at the time of diagnosis (gastric wall penetration and lymph node metastasis). Local recurrence may occur also in those patient which had R0 resection: management of these cases is extremely difficult for the involvement of regional structures resulting in poor surgical chances. Therefore multidisciplinary therapeutic approach is necessary to achieve better results. The aim of this report is to refer about an unusual presentation of local relapse in an old patient submitted to a total gastrectomy in which surgical approach permitted a good control of the disease.

## Case report

**Figure 1:**
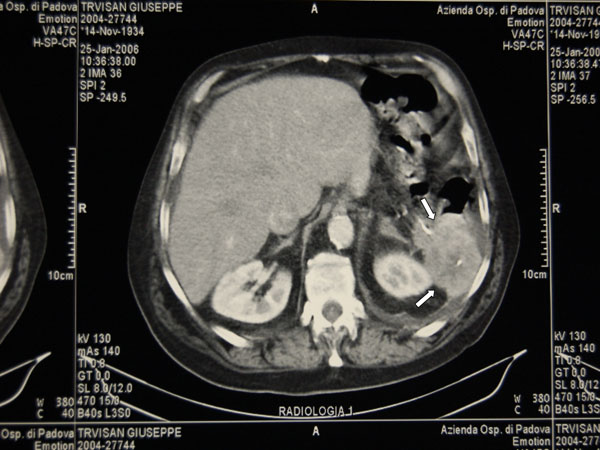
CT scan demonstrating a bulk in splenic area of about 8 cm in diameter and infiltrating pancreatic tail with adhesions to left diaphragm, left colic flexure and left kidney fascia

**Figure 2:**
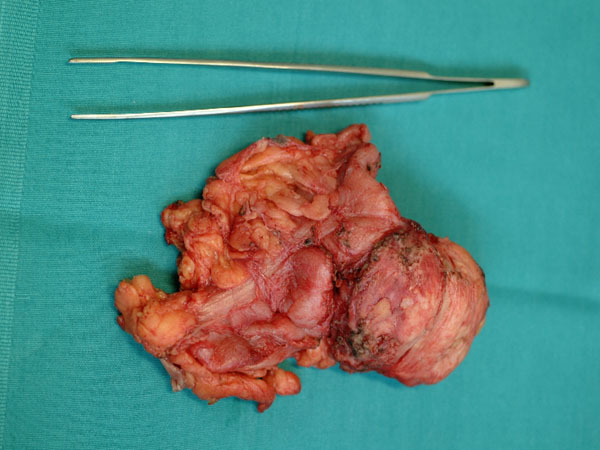
Surgical specimen comprehensive of the bulk resected en-bloc with pancreatic tail, left colic flexure, a portion of left diaphragm and kidney fascia.

Male, 71 years old; on July 27, 2004, he was submitted to total gastrectomy with splenectomy and lymph-node dissection (D2) for an ulcerated adenocarcinoma of the greater curvature of the upper third of the stomach. Roux-en-Y stapled esofago-jejunoanastomosis was performed and oral food intake resumed in 7^th^ post-operative day after X-ray control with hydro-soluble contrast. Hospital stay was prolonged by left basal pneumonia associated to pleural effusion; discharge occurred after one month. Histological examination demonstrated an adenocarcinoma (Laurèn intestinal type, Ming infiltrating type) extended to all layers of gastric wall and metastasis to greater curvature lymph-nodes (station 4 of JGCA) [1]; the others stations of JGCA (from 1 to 12, excluded 4) were non metastatic (43 lymph-nodes were examined); it was stage IIIA according to TNM classification (T3 N1 M0) [2]. After discharge chemotherapy was given (Etoposide/Leucovorin/5-fluorouracil, ELF-regimen) from September 2004 to March 2005. Follow-up was uneventful till December 2005. In this period patient suffered of left hypochondriac pain, mild dyspnoea and anorexia. Esophagojejunoscopy was unremarkable. X-Ray confirmed left basal pleural effusion; blood examination resulted only in CEA increase (19.3 ug/L). CT scan demonstrated a bulk in splenic area of about 8 cm in diameter and infiltrating pancreatic tail with adhesions to left diaphragm, left colic flexure and left kidney fascia (Fig. 1). On 7 February 2006, he was submitted to explorative laparotomy that confirmed CT scan report. The bulk was resected en-bloc with pancreatic tail, left colic flexure, a portion of left diaphragm and kidney fascia; bowel continuity was restored by side-to-side stapled anastomosis; diaphragm was directly sutured (Fig. 2). Accurate inspection of abdominal cavity excluded others localizations of the disease. Postoperative period was uneventful as regards surgical problems. Angina attack compared in 9^th^ post-operative day; after resolution of this complication patient was discharged. Histological examination resulted in poor differentiated gastric carcinoma infiltrating colic wall and pancreas with metastasis in one of nine peri-colic lymph-nodes examinated.

## Discussion

Loco-regional recurrence and distant metastases are common events after surgery for gastric adenocarcinoma. Abdominal extraluminal recurrence of gastric cancer is a disarmimg condition because of poor therapeutic chances. Generally it is a matter of peritoneal carcinosis or multiple liver metastasis; in these cases surgery has little opportunities to be useful. Literature reports rare cases of single localization in abdominal cavity that may be resected. Menzel [3] reported a case of infrarenal aortic aneurysm whose detection permitted to discover gastric carcinoma. A similar condition is reported by Shimoyama [4] who diagnosed gastric cancer after nephroureterectomy for hydronephrosis due to ureteral metastasis. Imachi [5] referred about metastatic adenocarcinoma to the uterine cervix. Rare extrabdominal localizations are reported: intramuscular gluteal tumour [6], scalp and forehead [7], testis [8], axillary lymph node [9]. Yoo and Colleagues [8] reported a multivariate analysis of risk factors involved in the recurrence of gastric cancer; in order they are lymph node metastasis, serosal invasion, infiltrative or diffuse type, larger tumour size (4 cm or greater), undifferentiated tumour and proximally located tumour. Serosal invasion and lymph node metastasis were common risk factors for all recurrence patterns. Buzzoni [10] underlined the role of radical surgery respect more conservative surgery to reduce the rate of loco-regional recurrence: particularly the pT stage was related to loco-regional recurrence whereas pN stage had importance on distant metastases. Motoori [11] developed a diagnostic system based on systematic analysis of gene expression profiling to predict the recurrence at clinically meaningful level: the prediction accuracy was high especially in patients with small tumours in I and II stage. Marrelli [12] obtained a scoring system with a regression model based on follow-up data to define subgroups of patients at risk for recurrence after radical surgery for gastric cancer. On the other hand, Bennet [13] affirmed that follow-up did not identify no symptomatic recurrence earlier than symptomatic one.

Our case is unusual in its presentation: an isolated bulk involving neighbouring organs suitable for surgical resection. The result after the en-bloc resection is very amazing. Considering the primary surgical specimen we may suppose the modalities of the local relapse: the spleen and local lymph-nodes were radically removed, but in spite of that, local contamination during the first operation remains the most reasonable interpretation. All risk factors suggested by Yoo and Colleagues [8] were present in the initial specimen: serosal invasion and nodal metastases, large tumour size (7 x 5 cm on the specimen) infiltrative and undifferentiated type and proximally located tumour. We can speculate that chemotherapy has favoured the delay of the clinical presentation of the recurrence. Recently developed new agents such as irinotecan, taxanes and capecitabine, provide more promising results also in metastatic gastric cancer such as new molecular targeting agents [14]. Encouraging perspectives may result from IORT by virtue of its technical properties which permits to exceed conventional doses [15]. We believe that an appropriate association between varies therapeutic option [16,17] (surgery, chemo and radiotherapy – EBRT and/or IORT-) may bring about a change for a better management of recurrent gastric cancer.

## Competing interests

The authors declare that they have no competing interests.

## Authors’ contributions

BM, CM and RL have studied the patient and performed the surgical operation. FC performed the endoscopic preoperative study of the patient and contributed to the literature review. CT contributed to the discussion of medico-legal issues and to the writing of the paper. BA and CM gave their contribution to the analysis of the data and contributed to the writing of the paper. All the authors read and approved the final manuscript.
